# Transfer of Nitrogen and Phosphorus From Cattle Manure to Soil and Oats Under Simulative Cattle Manure Deposition

**DOI:** 10.3389/fmicb.2022.916610

**Published:** 2022-06-14

**Authors:** Chengzhen Zhao, Juan Hu, Qiang Li, Yi Fang, Di Liu, Ziguang Liu, Rongzhen Zhong

**Affiliations:** ^1^Jilin Provincial Laboratory of Grassland Farming, Northeast Institute of Geography and Agroecology, Chinese Academy of Sciences, Changchun, China; ^2^School of Resources and Environment, University of Chinese Academy of Sciences, Beijing, China; ^3^Key Laboratory of Combining Farming and Animal Husbandry, Ministry of Agriculture, Animal Husbandry Research Institute, Heilongjiang Academy of Agricultural Sciences, Harbin, China

**Keywords:** cattle manure deposition, nutrient return, root length, bacteria, fungi

## Abstract

Simulated cattle manure deposition was used to estimate nutrient transfer to soil and oats and to investigate changes in microbial community composition and functional groups in oat rhizospheres. Nutrient absorption and return efficiency were calculated as a series of standard calculation formulas, and total nutrient transfer efficiency was nutrient absorption efficiency plus nutrient return efficiency. In total, 74.83% of nitrogen (N) and 59.30% of phosphorus (P) in cattle manure were transferred to soil and oats, with 11.79% of N and 7.89% of P in cattle manure absorbed by oats, and the remainder sequestered in the soil for 80 days after sowing. Cattle manure increased oat root length, surface, and volume under 0.2 mm diameter, and improved relative abundance of the microbiome known to be beneficial. In response to cattle manure, several bacteria known to be beneficial, such as Proteobacteria, Bacteroidota, and Firmicutes at phyla the level and *Pseudoxanthomonas*, *Pseudomonas*, and *Sphingomonas* at the genus level, were positively related to oat biomass and nutrient accumulation. For fungal communities, the relative abundance of Ascomycota is the predominant phylum, which varied in a larger range in the control treatment (81.0–63.3%) than the cattle manure deposition treatment (37.0–42.9%) as plant growing days extend. The relevant abundance of Basidiomycota known as decomposer was higher in cattle manure deposition treatment compared to that in control treatment at 15 days after sowing. More importantly, cattle manure deposition inhibited trophic mode within pathotroph like *Alternaria* and *Fusarium* fungal genus and promoted saprotroph and symbiotroph.

## Introduction

In native grassland ecosystems, soil N and P are absorbed to support plant growth, and 30–50% of above-ground plant biomass is consumed by grazing animals (e.g., cattle), with portions of N and P returned to the soil in excreted wastes (Yoshitake et al., 2014). Appropriate application of animal manure and its subsequent decomposition provide highly plant-available nutrients that stimulate plant production (Van der Waal et al., 2011). In cattle feces, the N concentration ranges from 18.0 to 26.2 g kg^–1^ (dry matter basis; Sordi et al., 2014), with 80% of total consumed plant N subsequently deposited on soil in cattle feces (Cardenas et al., 2016). Although the amount of N and P from plant to cattle is easily evaluated, the amount of N and P from cattle manure to soils is not as well evaluated. Appropriate application of cattle manure to grasslands can maintain soil quality (Pätzhold et al., 2013), whereas excessive applications cause substantial reactive N and P, with volatilization and environmental contamination (Kumar et al., 2017).

Large amounts of organic matter from cattle dung provide energy sources and beneficial rhizospheric conditions for the growth of the microbiome (Chaparro et al., 2012; Poeplau and Don, 2015). In this situation, soil microbiome degrades macromolecular organic matters, promoting microbial nutrient recycling and energy flowing in terrestrial ecosystems (Rosinger et al., 2019). These changes in grassland promoted the catalyzers of nutrient cycling such as carbon sequestration (Smith et al., 2015), ammonia oxidation (Prosser and Nicol, 2012), and phosphorus transformation (Parniske, 2008).

Rhizosphere microbes are closely associated with soil nutrition and plant growth (Kumar et al., 2016). Microbial communities are species-specific in the rhizosphere environment, as plant roots acquire specific microorganisms from soil (Hein et al., 2008). These species-specific microorganisms have high functional diversity (Escalas et al., 2019) and drive soil functions at specific time points (Bastida et al., 2016). Plant rhizosphere microbial communities are influenced by numerous factors, mainly plant development (Schlemper et al., 2017) and soil nutrient status (Meena et al., 2017). As plants develop, they release specific metabolites to select microorganisms, presumably to support specific functions (Chaparro et al., 2014; Zwetsloot et al., 2020) and perhaps in association with changes in nutrient requirements. For example, legumes release flavones to enhance rhizobia-legume symbiosis as plants usually require more N later in development (Zhang et al., 2009). However, it was previously reported that microbial communities are modified by fertilization, with decreased dependence of the microbiome on root exudates (Ai et al., 2015; Zhao et al., 2019, 2020). Cow manure provides the main source of energy for microbes as it contains a lot of labile carbon that can stimulate microbial aggregation, activity, and diversity (Delgado-Baquerizo et al., 2016). Subsequently, the microbiome decomposed the organic matter in cow manure and increased nutrient availability (Jacoby et al., 2017).

Applying cattle manure and incorporating it into the soil has been widely investigated in agriculture ecosystem (Sha et al., 2012). Rhizosphere microbes, e.g., autotrophic archaea oxidize ammonium (NH4^+^) to nitrate (NO3^–^) by nitrification (Leininger et al., 2006; Chen et al., 2015), whereas the rhizosphere core microbiome, *Acidobacteria* and *Sphingobacteriales*, are involved in N cycling (Hester et al., 2018). Arbuscular mycorrhizal fungi (AMF) can access P even in root P-depletion zones (Walder and Heijden, 2015). Moreover, plant beneficial rhizospheric microorganisms or plant growth-promoting microorganisms, such as *Azospirillum, Enterobacter, Pseudomonas, Klebsiella, Serratia*, and *Pantoea* species, were associated with plant growth in a variety of grass species (Lange et al., 2015). The surface application of cattle manure affects functional microbes, e.g., autotrophic and heterotrophic nitrifiers (Wu et al., 2020). Applying cattle manure to grassland soils can cause rapid changes to the availability of N and P (Geisseler and Scow, 2014) which stimulate microbes, especially those related to ammonia oxidation (Mooshammer et al., 2014) and phosphorus transfer (Richardson et al., 2009). However, fecal N could be lost because of ammonia volatilization and leaching during the growing season (Maris et al., 2021). Ammonia volatilization is the main loss pathway for N from cattle dung deposited on grassland influenced by multiple factors such as temperature, rainfall, and wind speed (Fischer et al., 2015). Generally, dry weather conditions will increase the volatilization of N (Meisinger and Jokela, 2000). Besides, rainfall increases t N leaching and subsequent denitrification of nitrate (NO_3_^–^) (Barger et al., 2013). Characterizing dynamics of microbial community composition, especially specific functional microbial groups, in response to the application of cattle manure, may provide new knowledge to optimize plant growth at various stages.

The root system connects the plant and soil and enables the absorption of nutrients and water in grassland ecosystems (Zhou et al., 2018). Roots are covered by highly complex rhizosphere microorganisms (Wilson, 2014). Rhizosphere, defined as the volume around living roots, is one of the most dynamic zones for nutrient influx (Sasse et al., 2017). As roots develop, vigorous root branching, high specific root length, and increased root length occupy a larger volume of soil, greatly increasing the rhizosphere and microbial activity (Sasse et al., 2017). The root system is relatively plastic and can change in response to soil fertility gradients that vary in time and space (Correa et al., 2019). Root functional traits, including root diameter (RD), root length (RL), root surface area (SA), root volume (RV), specific root length (SRL), and root tissue density (RTD), are affected by fertilizer applications in agriculture ecosystems (Kramer-Walter et al., 2016; Wen et al., 2019). However, responses of root traits to the application of cattle manure in grassland ecosystems are poorly understood (Carmona et al., 2013). Understanding the response of root morphology to the application of manure should improve understanding of nutrient transfer and predictions of plant production.

Oat (*Avena sativa* L.) is a very important forage in animal husbandry in China as it has high yield and nutritive value. From 2015 to 2017, the imported oat grass increased from 0.15 million tons to 0.30 million tons. Besides, from 2012 to 2015, the planting area increased from 21,800 hm^2^ to 334,200 hm^2^, with the output of oat increasing from 1.8416 million tons to 2.7436 million tons (Guo T. et al., 2019). However, demand for oat is high in China, and dairy farming alone requires a minimum of about 1.75 million tons of quality oat at present (Guo T. et al., 2019). In China, oat grass is fed to cattle and the resulting manure is returned to the grassland which was a new mode of planting and breeding circular agriculture in recent years.

In the present study, by using oats (*A. sativa* “Baiyan No.2”) growing in pots as a model of cattle manure application to oat grassland, the objectives of this study were to: (1) estimate the transfer of N and P from manure to soils; (2) investigate microbial community dynamics, especially specific functional microbial groups in response to cattle manure; and (3) determine root dynamical morphology changes in response to available nutrients.

## Materials and Methods

### Plant Growth Conditions and Experimental Design

A pot experiment was performed from July to September 2019 at an automatic greenhouse at the Agricultural Ecology Station of Northeast Institute of Geography and Agroecology Chinese Academy of Sciences, in Changchun City, Jilin province (43°59′54′ N, 125°23′57′E). This site has a temperate continental monsoon climate. The mean annual temperature is 6.4°C, and the mean annual precipitation is 614 mm. This soil is classified as typical thin black soil. The soil is a clay loam (Typic Hapludoll, USDA Soil Taxonomy) with an average of 36.0% clay, 24.5% silt, and 39.5% sand. In this area, the maize crop was planted under conventional tillage management since 2012. Soil (0–25 cm depth) was collected from a farmland near the greenhouse. After passage through a 2-mm sieve, the soil was well mixed and packed into 24 pots (20 cm diameter and 25 cm high) with 6 kg soil per pot (1.21 g cm^–3^ bulk density). Each pot was sown with 2.0 g of oat seeds (50 seeds pot^–1^) on July 1. After sowing, 12 pots were covered with 0.5 kg fresh cattle manure from a cattle fattening farm, as simulated cattle manure deposition (CMD) treatment, whereas cattle manure was not applied to 12 pots and they were the controls (CON). The basic properties of soil and fresh cattle manure are shown (Supplementary Table 1). Plants were grown under a photoperiod of 16 h light (from 6 am to 10 pm), followed by 8 h dark, at a light intensity of 200 ± 20 μmol m^–2^ s^–1^ (LED-T8) and 50–75% RH, with an average temperature of 25/18°C (day/night) (Wen et al., 2019). An artificial watering method (three times per week) was used, with equal volumes of water for all pots.

### Sample Collection and Measurement

At the 0 days (July 1, sowing date), six soil samples were collected before packing pots as the initial soil and were sifted through a 2-mm sieve and homogeneously mixed. Approximately 6 g of each initial soil sample was put into a 5-ml PE tube and placed in liquid N for microbial sequencing, whereas the remainder was air-dried at room temperature for 1 week and used for chemical analyses. During growth, three random pots in the control treatment and cattle manure deposition treatment were collected for shoot, root, and bulk soil samples on the following days: 15 days after sowing (July 16, trefoil stage), 47 days after sowing (August 17, elongation stage), 66 days after sowing (September 5, pustulation stage), and 80 days after sowing (September 19; maturity stage). The rhizosphere soil (soil firmly attached to roots) of each pot was only collected 15 days after sowing and 66 days after sowing. Briefly, at each sampling, all shoots of each pot were separated from below-ground parts, dried at 65°C for 48 h in a forced-air drying oven, and weighed to determine the total above-ground biomass (AB). Subsequently, all shoot samples were shaken on a 1-mm sieve and stored at 4°C for subsequent chemical analyses. Then, the complete root system of each pot was carefully extracted by removing most soil around the root system. The remaining fine roots in the soil were collected using forceps to minimize soil losses. Then, complete roots and fine roots were washed in a mesh bag (1 μm; 10 cm × 10 cm × 25.5 cm) and stored at 4°C prior to analysis. Finally, the bulk soil of each control treatment pot was well mixed before sampling, whereas for cattle manure deposition treatment pots, surface cattle manure was first removed and then the bulk soil was well mixed and sampled. All soil samples were passed through a 2-mm sieve and air-dried before chemical analysis. For rhizosphere soil sampling at trefoil and pustulation stages, rhizosphere soil from each pot was collected before sampling root by gently shaking the whole plant root system to remove loosely attached soil, and then the soil adhering to the root system was placed into two replicated 5-ml PE tubes. Rhizosphere soil samples were immediately transferred into liquid N and stored at −80°C prior to microbial sequencing.

For root morphology analysis, all roots for each control treatment or cattle manure deposition treatment pot were spread out in water on a glass tray to reduce root overlap and scanned using a flatbed scanner (EPSON Perfection V700 Photo, Seiko Epson Corp., Japan) at a resolution of 600 dpi, as described (Bouma et al., 2001). Subsequently, all roots of each control treatment or cattle manure deposition treatment pot were oven-dried at 65°C for 48 h and weighed as below-ground biomass (BB). Images were saved in an uncompressed TIFF format. RL, SA, and V at various RD classes (<0.2, 0.2–0.5, 0.5–1, 1–2, and >2.0 mm) were measured by WinRHIZO image analysis software (Epson 1680, WinRHIZO Pro2003b, Regent Instruments Inc., Quebec, Canada). Other traits, including the SRL (the ratio of the root length to its BB) and the RTD (the ratio of BB to its fresh volume) were calculated.

Soil organic carbon (SOC) was analyzed by the K_2_Cr_2_O_7–_H_2_SO_4_ oxidation–reduction colorimetric method (Yeomans and Bremner, 1988). Total N (TN) and phosphorus (TP) of soil were measured by Kjeldahl digestion and colorimetric analysis, respectively (Institute of Soil Science, Chinese Academy of Sciences [ISSCAS], 1978). Soil available nitrogen (AN) and phosphorus (AP) were measured by alkaline diffusion and NaHCO_3_ extraction (0.5 mol L^–1^, pH 8.5), respectively (Bao, 2000; Liang et al., 2014). For the plant, above-ground N content (ANC) was measured by N Kjeldahl, and above-ground P content (APC) was measured colorimetrically at 880 nm after reaction with molybdenum blue, as well as below-ground N and P contents (BNC and BPC). Two samples from each pot were analyzed and averaged.

Nutrient absorption and return efficiency were calculated as follows: Above-ground N or P accumulation (ANA and APA, g pot^–1^) = AB × ANC or AB × APC, respectively. The same calculation was used for below-ground N and P accumulation content (BNA and BPA, g pot^–1^). N absorption efficiency (NAE,%) = [(ANA + BNA in cattle manure deposition treatment pot) - (ANA + BNA in control treatment pot)]/amount of nutrient in cattle manure × 100%. The same calculation was used for P absorption efficiency (PAE,%). Furthermore, N return efficiency (NRE,%) from feces to soil = [(TN in each cattle manure deposition treatment pot) – (TN in each control treatment pot)]/amount of N in cattle manure × 100%, with the same calculation used for P return efficiency (PRE,%). Total N and P transfer efficiency (TNE and TPE) from feces to soil and oat = NAE + NRE and PAE + PRE, respectively.

### DNA Extraction and Illumina High-Throughput Sequencing

To extract soil microbial DNA from 0.5 g rhizosphere soil samples, a FastDNA™ SPIN Kit was used according to the manufacturer’s protocol (MP Biomedicals, Santa Ana, CA, United States). The DNA was diluted to 1 ng μl^–1^ and stored at −20°C pending analysis. Bacterial 16S and fungal ITS rRNA gene sequences were measured to characterize microbial populations. For bacterial diversity analysis, the primers 515F (5′-GTGCCAGCMGCCGCGGTAA-3′) and 806R (5′-GGACTACHVGGGTWTCTAAT-3′) were used to amplify the flexible V4 region of the 16S rRNA gene. For fungal diversity analysis, the primers ITS1-1F-F (5′-CTTGGTCATTTAGAGGAAGTAA-3′) and ITS1-1F-R (5′-GCTGCGTTCTTCATCGATGC-3′) were used to amplify the ITS1-1F region.

The reaction mix (30 μl) contained 1.5 μl of each primer, 10 μl of template DNA (1 ng μl^–1^), 15 μl of Phusion High-Fidelity PCR Master Mix (BioLabs, Inc., Waltham, MA, United States), and 2 μl of water. The thermal program included 25 cycles of 98°C for 30 s, 50°C for 30 s, and 72°C for 1 min. PCR amplicon quality was evaluated by gel electrophoresis and the amplicons were purified using Qiagen Gel Extraction Kit (Qiagen, Hilden, Germany). Amplicons from each sample were amplified in the second round of PCR, purified with a Qiagen Gel Extraction Kit, and quantified using a Qubit dsDNA Assay Kit.

Equimolar amounts of purified amplicons were pooled and subjected to high-throughput sequencing on a HiSeq platform (Illumina, San Diego, CA, United States) at Beijing Biomarker Corporation. Raw paired-end reads were quality-filtered with Trimmomatic software (version 0.33; Bolger et al., 2014), and FLASH software (version 1.2.11) was used to joint for paired reads, and potential chimeras were removed using UCHIME (version 8.1) (Edgar et al., 2011) as implemented in MOTHUR (Schloss et al., 2009). QIIME software (version 1.90) was used to analyze sequences (Caporaso et al., 2010). Finally, UPARSE pipeline was used to classify the operational taxonomic units (OTUs) with 97% similarity UCLUST software Version 1.2.22 (Edgar, 2013). SILVA (Quast et al., 2013) was used for taxonomic annotations of bacterial and fungal OTUs and a minimum similarity cutoff of 90% was used for conservative OTU assignments. The data evaluation of sample sequencing for bacteria (Supplementary Table 2) and fungi (Supplementary Table 3) and multi-samples’ rarefaction curves for bacteria (Supplementary Figure 1) and fungi (Supplementary Figure 2) demonstrated that our sequencing data represented most of their compositions. Ecological and metabolic functions were predicted for bacterial and fungal OTUs using Functional Annotation of Prokaryotic Taxa (FAPROTAX; Louca et al., 2016) and fungi functional guild (FunGuild), respectively (Nguyen et al., 2016). The sequencing reads of the bacterial 16S rRNA gene and fungal ITS rDNA gene were deposited in the Sequence Read Archive of the National Center for Biotechnology Information (NCBI) under the accession numbers PRJNA830643 and PRJNA831303, respectively.

### Statistical Analyses

Independent Student’s *t*-tests were done to assess the effects of cattle manure deposition on all parameters separately for each plant growth stage. Two-way analyses of variance (ANOVA) were used to analyze the effects of treatment (cattle manure deposition or control) and plant growth stage (plant trefoil stage and plant pustulation stage) on plant and soil parameters, and on nutrient absorption and return efficiency. Non-metric multidimensional scaling (NMDS) of the bacterial and fungal communities based on OTU composition was done using the Bray–Curtis similarity metric to identify differences between cattle manure deposition treatment and control treatment microbial communities at each sampling time. Differences in bacterial and fungal communities between treatments were tested by the analysis of similarities (ANOSIM and ADONIS). Relative abundance at phylum and genus levels and each specific bacterial function and fungal guild in a given sample was calculated as a percentage value by dividing the raw number of sequences associated with the specific taxa by the total number of sequences in the sample. Positive or negative relationships between environmental factors (nutrient absorption and return efficiency, root traits, and soil properties) and microbial communities (bacterial and fungal at phyla and genus level, respectively) were examined using canonical correspondence analysis (CCA). For all parameters (plant and soil parameters, root traits, and rhizosphere soil microbial sequencing), two samples from each pot were analyzed and averaged and three replicates (pots) were used for data analysis. All parameters were analyzed with SPSS version 16.0 (SPSS Inc. Chicago, IL, United States), with *P* < 0.05 considered significant. All figures were generated by Origin 2016 Pro.

## Results

### Fecal N and P Transfer

There was an interaction (*P* < 0.001) between cattle manure deposition treatment and plant growth stages on above- and below-ground biomass. Briefly, compared to control treatment, above- and below-ground biomass of oat in cattle manure deposition treatment at 80 days after sowing increased 1.9 and 1.5 times, respectively, whereas above- and below-ground biomass N and P contents increased 19–57%. Consequently, both above and below-ground N and P accumulation were 2.1 to 2.9 higher in response to cattle manure deposition treatment compared with the control treatment (Table 1). N and P absorption efficiency increased (*P* < 0.001) with the mature stage extension. Finally, 11.79% of total N and 7.89% of total P in cattle manure were absorbed by oats during the entire growth period, of which 95% of absorbed N and P was deposited to the above-ground part of oats.

**TABLE 1:** The dynamics of N and P transfer from cattle manure to soil and oats along plant growth stage under cattle manure deposition.

Items	Treatment								*P* - value			
	15 days after sowing		47 days after sowing		66 days after sowing		80 days after sowing		SEM	GS	CMD	GS*CMD
	CON	CMD	CON	CMD	CON	CMD	CON	CMD				
Biomass (g/pot)												
Aboveground biomass	2.33	2.97*	9.93	16.37*	16.70	28.60*	17.73	32.77*	2.167	*P* < 0.001	*P* < 0.001	*P* < 0.001
Belowground biomass	0.45ns	0.43	0.73	1.61*	0.97	1.73*	1.26	1.89*	0.114	*P* < 0.001	*P* < 0.001	*P* < 0.001
C return												
Soil organic carbon (g.kg)	12.47	14.82*	11.57	15.68*	11.13	15.91*	10.87	16.23*	0.319	0.873	*P* < 0.001	*P* < 0.001
N absorption and return												
Aboveground N content (g.kg)	26.32	35.83*	19.83	20.77ns	13.21	15.57*	11.76	14.01*	1.127	*P* < 0.001	*P* < 0.001	*P* < 0.001
Belowground N content (g.kg)	13.36	16.16*	11.34	12.63ns	7.99	12.55*	8.01	12.45*	0.401	*P* < 0.001	*P* < 0.001	0.002
Aboveground N accumulation (g pot^–1^)	0.061	0.106*	0.197	0.342*	0.221	0.445*	0.208	0.459*	0.029	*P* < 0.001	*P* < 0.001	0.001
Belowground N accumulation (mg pot^–1^)	5.97	6.89ns	8.33	20.37*	7.72	21.66*	10.08	23.59*	1.482	*P* < 0.001	*P* < 0.001	*P* < 0.001
Soil total N content (g.kg)	0.894	0.907ns	0.887	1.002ns	0.874	1.082*	0.862	1.097*	0.023	0.304	*P* < 0.001	0.095
Soil available N content (mg.kg)	100.6	244.4*	109.9	256.3*	174.9	297.6*	78.2	247.6*	14.684	0.058	*P* < 0.001	0.875
P absorption and return												
Aboveground P content (g kg)	5.58	7.22*	3.77	5.24*	3.00	4.30*	2.20	3.46*	0.239	*P* < 0.001	*P* < 0.001	0.912
Belowground P content (g kg)	3.34	5.40*	2.30	2.57ns	1.83	2.40*	1.63	2.25*	0.170	*P* < 0.001	*P* < 0.001	*P* < 0.001
Aboveground P accumulation (g pot^–1^)	0.013	0.021*	0.037	0.086*	0.050	0.123*	0.039	0.113*	0.008	*P* < 0.001	*P* < 0.001	0.001
Belowground P accumulation (mg pot^–1^)	1.49	2.30ns	1.69	4.14*	1.77	4.15*	2.05	4.27*	0.250	*P* < 0.001	*P* < 0.001	0.015
Soil total P content (g kg)	0.351	0.362ns	0.348	0.386*	0.343	0.405*	0.341	0.425*	0.005	*P* < 0.001	*P* < 0.001	*P* < 0.001
Soil available P content (g kg)	13.47	16.51*	12.18	17.75*	13.72	19.08*	9.60	16.63*	0.446	*P* < 0.001	*P* < 0.001	*P* < 0.001
Nutrient absorption efficiency (%)												
N absorption efficiency		2.05c		7.00b		10.58a		11.79a	1.229		0.001	
P absorption efficiency		0.94c		5.14b		7.60ab		7.89a	0.906		0.001	
Nutrient return efficiency (%)												
N return efficiency		3.67c		30.95b		55.88a		63.05a	5.316		*P* < 0.001	
P return efficiency		6.20d		23.15c		37.45b		51.41a	3.619		*P* < 0.001	
Total nutrient transfer efficiency (%)												
Total N transfer efficiency		5.72c		37.95b		66.46a		74.83a	8.439		*P* < 0.001	
Total P transfer efficiency		7.14d		28.29c		45.05b		59.30a	5.905		*P* < 0.001	

GS, growth stage. 15 days after sowing corresponds to the plant trefoil stage; 47 days after sowing corresponds to the plant elongation stage; 66 days after sowing corresponds to the plant pustulation stage; 80 days after sowing corresponds to the plant maturity stage. CON, control; CMD, cattle manure deposition. Data represents mean ± SEM (n = 3). The data was based on a pot experiment in one year. All parameters were analyzed with SPSS version 16.0 (SPSS Inc. Chicago, IL, United States). For biomass, C return, N and P absorption and return, significant differences are indicated (* represents P < 0.05). For N and P absorption and return efficiency, within a growth stage, means without a common superscript differed (P < 0.05).

The SOC had a decreasing tendency in control treatment but an increasing tendency in cattle manure deposition treatment as oat development, with similar variation tendency in TN and TP (Table 1). Thus, cattle manure deposition treatment increased 49% SOC (*P* < 0.001), 27% TN (*P* < 0.001), and 25% TP (*P* < 0.001) of soil when compared to control treatment. Moreover, both N and P return efficiency in cattle manure deposition treatment increased (*P* < 0.001) as oats developed, with 63.05% of fecal N and 51.41% of fecal P returned to the soil throughout the whole growth period.

### Responses of Root Traits to Cattle Manure Deposition

**FIGURE 1:**
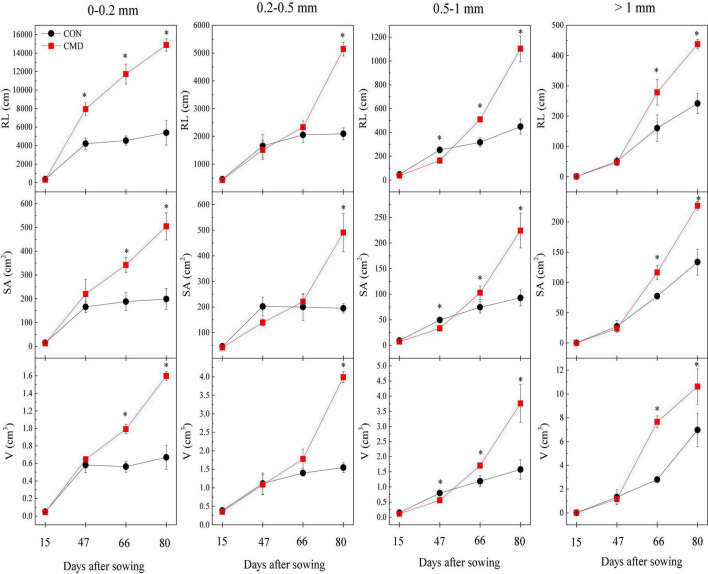
Root length (RL), surface area (SA), and volume (V) at <0.2, 0.2–0.5, 0.5–1, and >1 mm root diameter of oat in CON and CMD treatments, at various growth stages. 15 days after sowing corresponds to the plant trefoil stage; 47 days after sowing corresponds to the plant elongation stage; 66 days after sowing corresponds to the plant pustulation stage; 80 days after sowing corresponds to the plant maturity stage. CON, control; CMD, cattle manure deposition. Data represents mean ± SE (*n* = 3). The data was based on a pot experiment in one year. All parameters were analyzed with SPSS version 16.0 (SPSS Inc. Chicago, IL, United States). Differences in each root trait whining different diameter classes between CON and CMD treatments at various growth stages are indicated (*represents *P* < 0.05).

**FIGURE 2:**
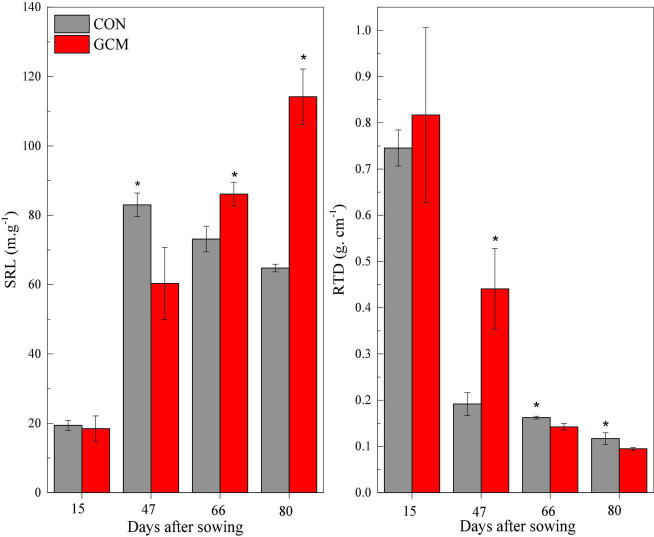
Specific root length (SRL) and root tissue density (RTD) of oat in CON and CMD treatments at various stages. 15 days after sowing corresponds to the plant trefoil stage; 47 days after sowing corresponds to the plant elongation stage; 66 days after sowing corresponds to the plant pustulation stage; 80 days after sowing corresponds to the plant maturity stage. CON, control; CMD, cattle manure deposition. Data represents mean ± SE (*n* = 3). The data was based on a pot experiment in one year. All parameters were analyzed with SPSS version 16.0 (SPSS Inc. Chicago, IL, United States). Differences between CON and CMD treatments in each root trait at various growth stages are indicated (* represents *P* < 0.05).

The root length of oats at <0.2 mm diameter increased (*P* < 0.01) in cattle manure deposition treatment compared with the control treatment for all plant growth stages, except trefoil stage (Figure 1). The root length, surface area, and volume of oat root at 0.2–0.5 mm diameter were higher (*P* < 0.01) in cattle manure deposition treatment than control treatment at 80 days after sowing. For oat roots at 0.5–1.0 mm diameter, the root length and surface area in cattle manure deposition treatment were lower (*P* < 0.05) at 47 days after sowing and then higher (*P* < 0.05) in cattle manure deposition treatment compared with the control treatment. As diameter exceeded 1 mm, root length and surface area at 47 days after sowing were not significantly affected by cattle manure deposition treatment, but increased (*P* < 0.05) afterward. The specific root length was decreased (*P* < 0.05) by cattle manure deposition treatment at 47 days after sowing and increased (*P* < 0.05) afterward, with the opposite for root tissue density (Figure 2).

### Response of Microbial Community Composition to Cattle Manure Deposition

**FIGURE 3:**
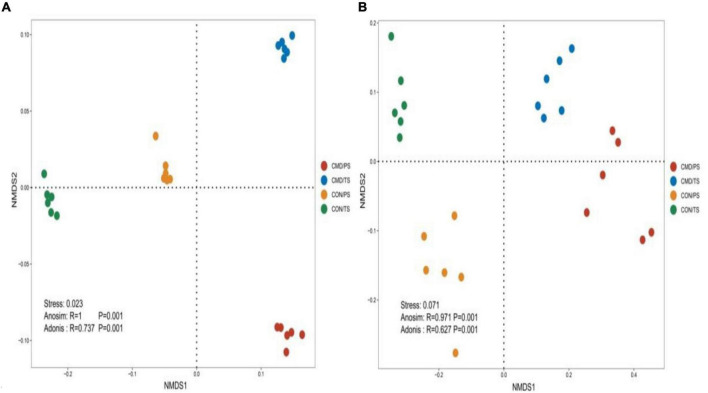
Non-metric multi-dimensional scaling (NMDS) of the bacterial **(A)** and fungal **(B)** community structures in CON and CMD treatments at various growth stages. TS, plant trefoil stage (15 days after sowing); PS, plant pustulation stage (66 days after sowing). CON, control; CMD, cattle manure deposition.

Non-metric multidimensional scaling ordination based on Bray–Curtis distances indicated that cattle manure deposition significantly affected the bacterial (Figure 3A) and fungal (Figure 3B) community composition.

**FIGURE 4:**
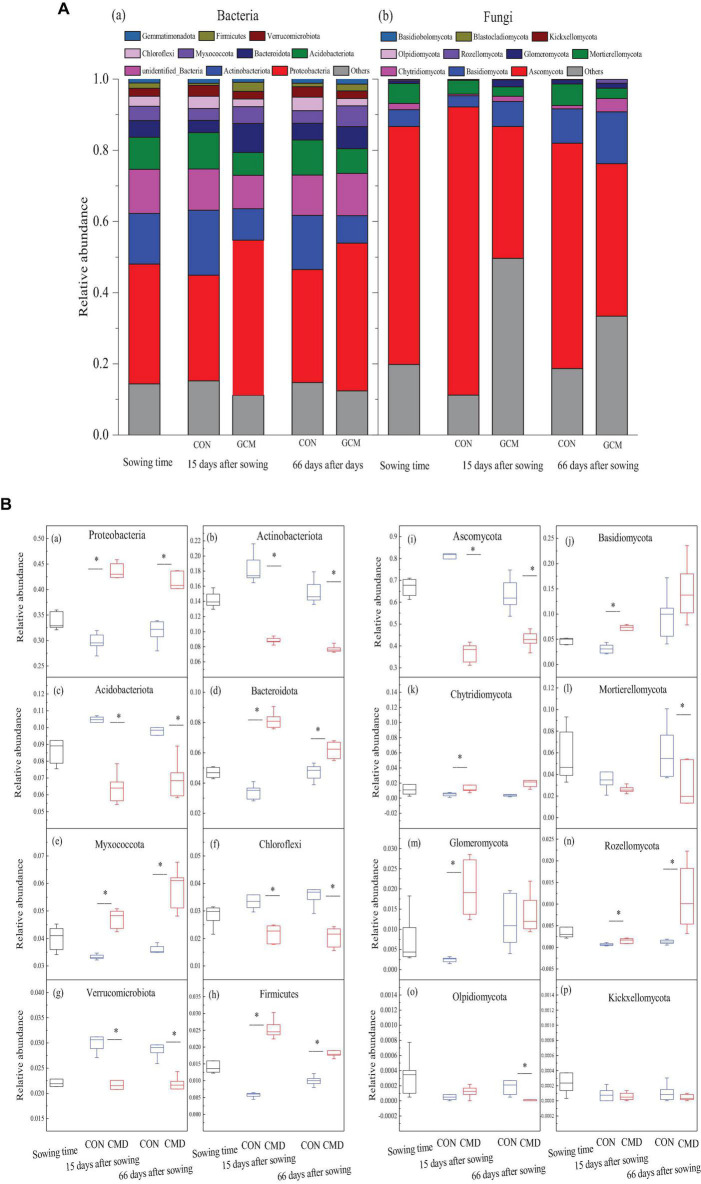
Bacterial and fungal community composition at phyla level under CON and CMD at various growth stages. **(A)** Taxonomic distribution of bacteria **(a)** and fungi **(b)** in different rhizosphere soil. **(B)** Relative abundance of the eight most dominant bacteria **(a–h)** and fungi **(i–p)** in different rhizosphere soil. 0 days corresponds to sowing date; 15 days after sowing corresponds to plant trefoil stage; 66 days after sowing corresponds pustulation stage. CON, control; CMD, cattle manure deposition. Data represents mean ± SE (*n* = 3). The data was based on a pot experiment in one year. All parameters were analyzed with SPSS version 16.0 (SPSS Inc. Chicago, IL, United States). Asterisk, on bar represent differences (*P* < 0.05) between CON and CMD treatments for bacteria at each growth stage.

Bacterial and fungal community structures at the phylum level under cattle manure deposition treatment and plant growth stage are shown in Figure 4A. Relative abundances of Proteobacteria, Firmicutes, and Bacteroidota in cattle manure deposition treatment at 15 and 66 days after sowing were higher (*P* < 0.01) than those in the control treatment, with a decreased tendency in cattle manure deposition treatment from 15 to 66 days after sowing. Moreover, cattle manure deposition treatment decreased (*P* < 0.001) the relative abundance of Actinobacteriota and Acidobacteriota at 15 and 66 days after sowing when compared to control treatment (Figures 4B a–h). Among fungal communities, Ascomycota was the predominant phylum, accounting for 37.0 and 81.0% relative abundance in cattle manure deposition treatment and control treatment, respectively at 15 days after sowing, and 42.9 and 63.3% relative abundance at 66 days after sowing. The relative abundance of Rozellomycota increased (*P* < 0.05) in cattle manure deposition treatment compared with control treatment at 15 and 66 days after sowing, whereas the relative abundance of Basidiomycota, Chytridiomycota, and Glomeromycota were higher (*P* < 0.05) in cattle manure deposition treatment at 15 days after sowing compared with the control treatment (Figures 4B i–p).

**FIGURE 5:**
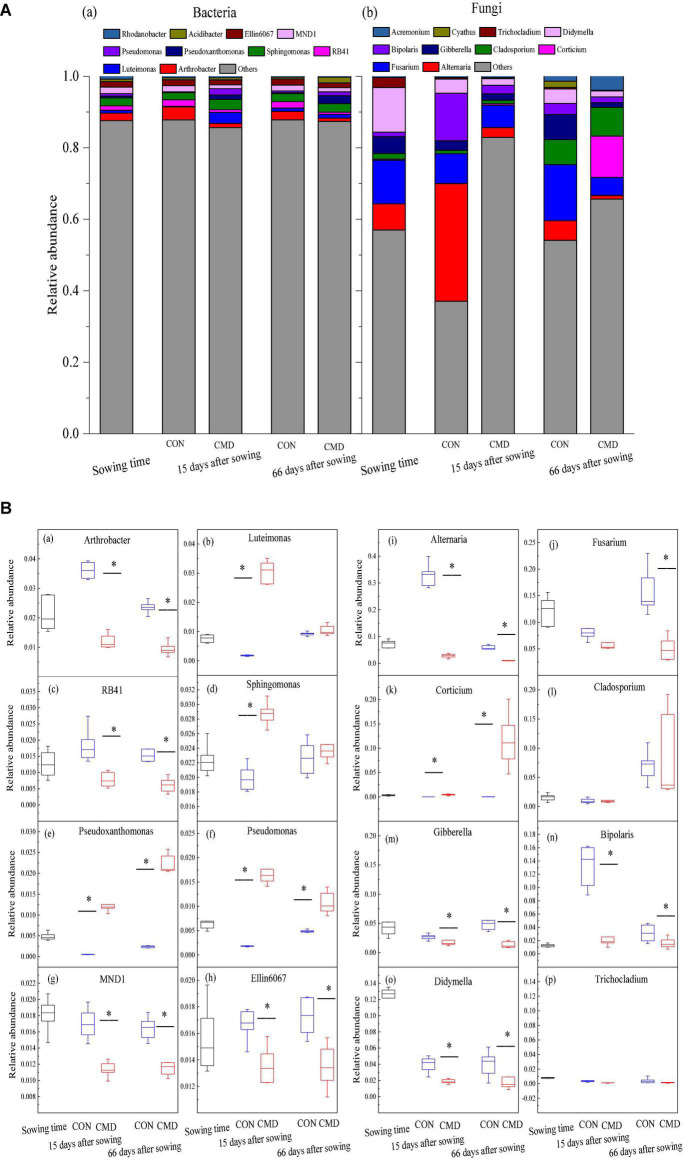
Bacterial and fungal community composition at genus level under CON and CMD at different growth stages. **(A)** Taxonomic distribution of bacteria **(a)** and fungi **(b)** in different rhizosphere soil. **(B)** Relative abundance of the eight most dominant bacteria **(a–h)** and fungi **(i–p)** in different rhizosphere soil. 0 days corresponds to sowing date; 15 days after sowing corresponds to plant trefoil stage; 66 days after sowing corresponds pustulation stage. CON, control; CMD, cattle manure deposition. Data represents mean ± SE (n = 3). The data was based on a pot experiment in one year. All parameters were analyzed with SPSS version 16.0 (SPSS Inc. Chicago, IL, United States). Asterisk, on bar represent differences (*P* < 0.05) between CON and CMD treatments for each microbe at each growth stage.

Bacterial and fungal community structures at genus level under cattle manure deposition treatment and plant growth stage are shown in Figure 5A. In the eight most frequently observed bacterial communities, relative abundances of *Pseudoxanthomonas* and *Pseudomonas* were higher (*P* < 0.01) in cattle manure deposition treatment versus control treatment at 15 and 66 days after sowing (Figures 5B a–h). For the fungal community, the relative abundance of fungi was observed, with either a decrease (*P* < 0.05) (*Alternaria*, *Fusarium*, *Gibberella*, and *Bipolaris*) or equal (*Cladosporium* and *Trichocladium*) in cattle manure deposition treatment compared with control treatment at 15 and 66 days after sowing (Figures 5B i–p).

### Responses of Microbial Functional Groups to Cattle Manure Deposition

**FIGURE 6:**
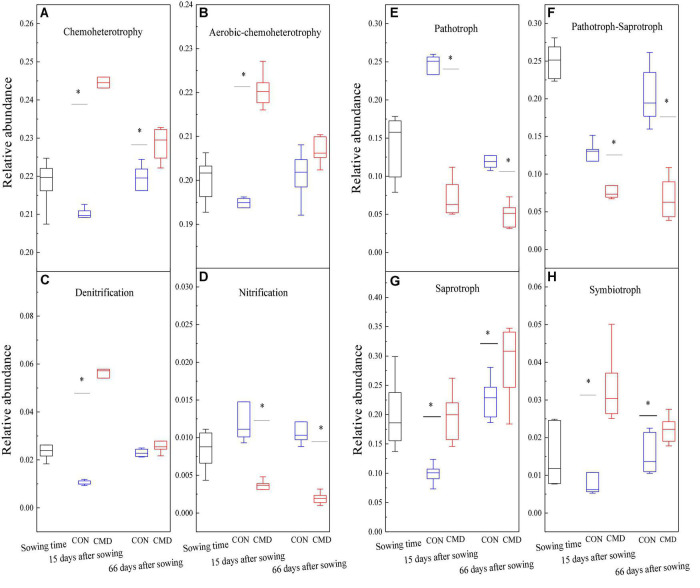
Bacterial **(A–D)** and fungal **(E–H)** functional groups based on FAPROTAX database and FUNguild database, respectively under CON and CMD treatments at different growth stages. CON, control; CMD, cattle manure deposition. 0 days corresponds to sowing date; 15 days after sowing corresponds to plant trefoil stage; 66 days after sowing corresponds pustulation stage. CON, control; CMD, cattle manure deposition. Data represents mean ± SE (n = 3). The data was based on a pot experiment in one year. All parameters were analyzed with SPSS version 16.0 (SPSS Inc. Chicago, IL, United States). Asterisk, on bar represent differences (*P* < 0.05) between CON and CMD treatments for each microbial functional group at each growth stage.

Chemoheterotrophy and aerobic chemoheterotrophy bacteria were decreased at 15 days after sowing in cattle manure deposition treatment versus control treatment (Figures 6A,B). Denitrification bacteria increased (*P* < 0.001) in the cattle manure deposition treatment at 15 days after sowing compared with control treatment, whereas nitrification bacteria decreased (*P* < 0.01) in cattle manure deposition treatment compared with control treatment at 15 and 66 days after sowing (Figures 6C,D). Among fungal functional groups, the relative abundance of Pathotroph and Pathotroph–Saprotroph were higher (*P* < 0.01) in the control treatment than cattle manure deposition treatment at 15 and 66 days after sowing (Figures 6E,F), whereas the relative abundance of saprotroph and symbiotroph were higher (*P* < 0.01) in cattle manure deposition treatment versus control treatment 15 days and 66 days after sowing (Figures 6G,H).

### Relationships Between Microbial Community Composition and Nutrient Absorption, Root Traits, and Nutrient Return

**FIGURE 7:**
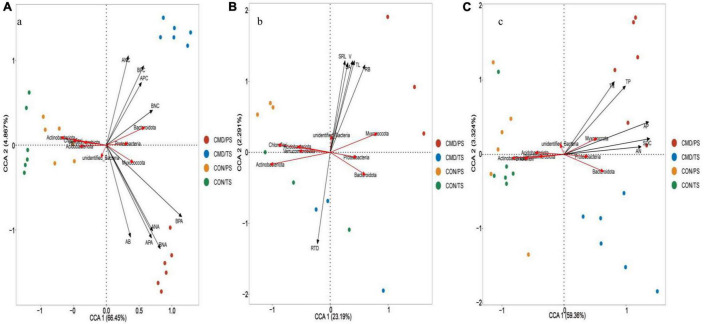
Canonical correspondence analysis (CCA) showing bacterial community composition at phyla level as affected by biomass and nutrient absorption and return **(A)**, and root traits **(B)** soil chemical property **(C)**. Microbial community composition located in the forward direction of the arrow of the environmental factor implies positive relationship between the microbial community composition and the environmental factor, and vice versa. TS, plant trefoil stage (15 days after sowing); PS, plant pustulation stage (66 days after sowing); AB, aboveground biomass; RB, below-ground biomass; ANC, above-ground N content; BNC, below-ground N content; APC, above-ground P content; BPC, below-ground P content; ANA, above-ground N accumulation; BNA, below-ground N accumulation; APA, above-ground P accumulation; BPA, below-ground P accumulation. CON, control; CMD, cattle manure deposition. RL, Root length; SA, root surface area; V, root volume, SRL, specific length; RTD, root tissue density; SOC, soil organic carbon; TN, total nitrogen; AN, available nitrogen; TP, total phosphorus; AP, available phosphorus. Data represents mean ± SE (n = 3). The data was based on a pot experiment in one year. All parameters were analyzed with SPSS version 16.0 (SPSS Inc. Chicago, IL, United States).

**FIGURE 8:**
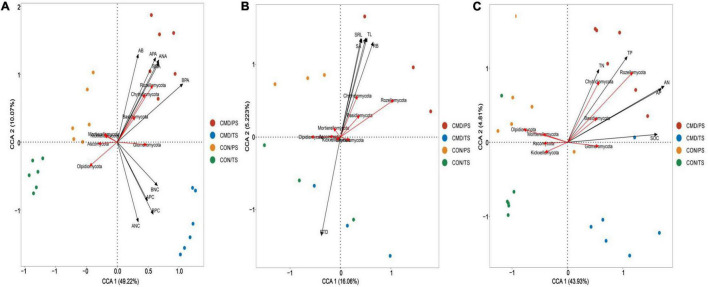
Canonical correspondence analysis (CCA) showing fungal community composition at phyla level as affected by biomass and nutrient absorption and return **(A)**, and root traits **(B)** soil chemical property **(C)**. Microbial community composition located in the forward direction of the arrow of the environmental factor implies positive relationship between the microbial community composition and the environmental factor, and vice versa. CON, control; CMD, cattle manure deposition.

Based on canonical correspondence analysis (CCA), bacterial and fungal community composition (phyla level) responded differently to changes in nutrient absorption, root traits, and nutrient return. The aboveground biomass, above- and below-ground N and P accumulation were the most crucial roles, with longer projection vectors than above- and below-ground N and P content in both bacterial and fungal community composition (Figure 7A). The above-mentioned parameters positively shaped Proteobacteria, Bacteroidota, and Myxococcota bacteria. The aboveground biomass and above- and below-ground N and P accumulation had strong positive impacts on Rozellomycota, Chytridiomycota, and Basidiomycota, whereas they had a strong negative impact on the Ascomycota in the fungal community (Figure 8A). Root biomass, root length, surface area, volume, and specific root length positively affected, and root tissue density negatively influenced Myxococcota bacteria (Figure 7B), whereas root biomass, root length, surface area, volume, and specific root length positively affected and root tissue density negatively influenced the Rozellomycota, Chytridiomycota, and Basidiomycota fungi (Figure 8B). Soil properties (SOC, TN, TP, AN, and AP) were intensely positively related to Myxococcota, Proteobacteria, and Bacteroidota bacteria and intensely negatively related to Actinobacteriota, Acidobacteriota, Chloroflexi, and Verrucomicrobiota bacteria (Figure 7C). In the fungal community, these parameters positively shaped the Rozellomycota, Glomeromycota, Chytridiomycota, and Basidiomycota, and negatively affected Ascomycota (Figure 8C).

Regarding microbial community composition at the genus level, aboveground biomass, above- and below-ground N and P accumulation had strong positive impacts on *Pseudoxanthomonas* bacteria, whereas above- and below-ground N and P content positively structured *Sphingomonas*, *Luteimonas*, and *Pseudomonas* bacteria (Supplementary Figure 3A). For fungal community, aboveground biomass, above- and below-ground N and P accumulation was positively related to *Corticium* and *Cladosporium* and negatively related to *Alternaria* and *Bipolaris* (Supplementary Figure 4A). Root biomass, root surface area, volume, and specific root length had positive effects, whereas root tissue density had negative effects on relative abundance of *Pseudoxanthomonas* bacteria (Supplementary Figure 3B). These root traits, with an exception of root tissue density, positively affected *Cladosporium* fungi but negatively influenced *Alternaria* and *Bipolaris* fungi (Supplementary Figure 4B). SOC, TN, TP, AN, and AP changed *Pseudoxanthomonas*, *Sphingomonas*, *Pseudomonas*, and *Luteimonas* bacteria and they had a strong negative impact on the Arthrobacter, *RB41*, *MND1*, and *Ellin6067* bacteria (Supplementary Figure 3C). In terms of fungal community, these soil properties were positively related to *Corticium* but negatively related to *Bipolaris*, *Alternaria*, *Fusarium*, *Didymella*, *Trichocladium*, and *Gibberella* (Supplementary Figure 4C).

## Discussion

### Fecal N and P Transfer

In grassland systems, cattle dung patches (0.1–1.75 kg) are an important nutrient source for soil and grass (Carmona et al., 2013; Rochette et al., 2014). In the present study, the transfer of fecal nutrients into soil and absorption by grassland plants were estimated using cattle manure deposition in a pot experiment. In previous studies, plants under cattle manure were temporarily inhibited due to the “smothering effect” of manure (Aarons et al., 2009), with growth stimulated by only 10-30 cm from the cattle dung edge (Yoshitake et al., 2014). However, in the present study, the application of cattle manure did not suppress oat growth, even at the trefoil stage or earlier. Cattle manure improved oat growth at all stages, especially in the late growth stage, in terms of increased biomass, nutrient absorption, nutrient accumulation, and nutrient absorption efficiency. In total, 11.79% of fecal N and 7.89% of fecal P were absorbed by oats.

Application of cattle manure on the soil surface returns fecal nutrients to soil depending on: (i) leaching the original available nutrients and (ii) rhizosphere microbial decomposition to release available nutrients (Jacoby et al., 2017; Jiao et al., 2019). When compared to control treatment, the SOC, TN, TP, AN, and AP of soil in cattle manure deposition treatment at 80 days after sowing were 49, 27, 25, 216, and 73% higher. In total, after the application of cattle manure, 74.83% of fecal N and 59.30% of fecal P were returned to the soil during the whole growing period. A portion of the N and P that returned to the soil were sequestered by oats, with a total of 63.05% of fecal N and 51.41% of fecal P left in the soil. Yoshitake et al. (2014) reported that 70% N of cattle manure entered the soil on a 30 × 100 m temperate area with a typical grazing intensity of ∼1.1 cattle ha^–1^ from late May to late October. However, the decomposition of cattle manure occurred slowly, requiring 350–850 days (Hirata et al., 2009). The weight of cattle manure decreased sharply to about 30% of the initial weight during the first 30 days under cattle manure deposition because of the proper temperature, high water content, and the activity of microbiome (Yoshitake et al., 2014). Another study showed that N of cattle manure in macro-mesh-size litterbags disappeared by 20% during 60 days and 17% during another 60 days (Rashid et al., 2017). In this study, the N transfer efficiency was increased and then decreased during 80 days after cattle manure deposition. N transfer efficiency was low at 3.67% during 15 days after cattle deposition and then increased to 27.28 and 24.93% between 15 days and 47 days after cattle deposition and between 47 days and 66 days after cattle deposition, respectively. However, the N transfer efficiency was only 8.37% between 66 days and 80 days after cattle deposition. P transfer efficiency had a similar tendency with N transfer efficiency.

In addition, losses of fecal N include ammonia volatilization and fecal AN leaching from fresh dung during the growing season (Maris et al., 2021). Consequently, not all fecal N is transferred into the soil during a single growing season. A better understanding of nutrient return by cattle manure deposition during the growing season will improve nutrient management decisions.

### Root Morphology

Root morphology was associated with nutrient absorption and plant biomass. Root growth is directly affected by the chemical properties of soil (Bandyopadhyay et al., 2010). More specifically, one of the most important nutrient-acquisition strategies of plants is thinner roots and larger specific root length (Wen et al., 2019). In the present study, root length of oats <0.2 mm in diameter was more susceptible than other root functional traits, and both root length and specific root length increased after elongation in cattle manure deposition treatment oats compared with the control treatment.

Roots have a close interaction with microorganisms in the rhizosphere. The presence of roots increased microbial abundance, activity, and variance (enrichment of select members or loss of detectable low-abundance taxa) by root exudates and strengthened the attachment of external nutrients to root systems (Landesman et al., 2019). As roots grow, the new tissue surface expands spaces for bacterial community, and the quick root tip elongation assembles more bacterial colonization on the root surface (Bulgarelli et al., 2012). For instance, phylum Proteobacteria are well-known rhizosphere colonizers and mainly positively respond to plant roots (Lundberg et al., 2012), consistent with present results that *Proteobacteria* was strongly positive to root biomass, root length, surface area, volume, and specific root length. Higher abundance of specific bacteria enriched in rhizosphere accelerated mineralization of organic matter and generated available nutrients around the root system, promoting root development (Valencia et al., 2018).

### Microbial Community Composition and Functional Groups

#### Bacterial Community Composition and Functional Groups

Plant growth stages filter and shape rhizosphere microbiomes community by microbial substrate preferences in root exudates (Shi et al., 2011). In addition, Proteobacteria, Actinobacteriota, and Firmicutes prefer easily decomposable substrates, such as sucrose, and consequently were reduced as the plant matured (Zwetsloot et al., 2020), as sucrose concentrations are higher in the early stages of plant growth and allocated primarily to the area behind the root tips (Schlemper et al., 2017). However, we only observed a relative abundance of Actinobacteriota in control treatment soil that distinctly decreased after the trefoil and postulation periods, whereas in Proteobacteria and Firmicutes it slowly increased.

Except for the effects of plant development stages, nutrient availability, especially soil N concentration, is the main driving force shaping the root microbiome community (Qu et al., 2020). Relative abundances of Proteobacteria and Firmicutes of rhizosphere soil increased as soluble organic N concentration increased (Gastal and Lemaire (2002). In this study, cattle manure significantly changed the relative abundance of dominant bacteria and remodeled bacterial variation tendency as plant development proceeded. In particular, manure greatly promoted relative abundance of Myxococcota, slightly promoted relative abundance of Proteobacteria, Firmicutes, and Bacteroidota, and intensely suppressed relative abundance of Actinobacteriota, Acidobacteriota, and Chloroflexi. Differences between cattle manure deposition treatment and control treatment in bacterial species may have been due to differences in adaptation and tolerances to nutrients (Coleman-Derr et al., 2016). In addition, more available nutrients at the late growing stage under cattle manure deposition treatment conditions may have suppressed bacterial growth. Supplementing N likely increased the amount of available nutrients for microbial growth if concentrations were below the threshold (Wei et al., 2013). However, soil N had the greatest impact on the microbial community and additional N suppressed both bacterial and fungal growth (Zhong et al. (2015), implying a potential N threshold for soil microbes in farmland ecosystems. The CCA in this study also supported this assumption, as soil AN and AP had positive impacts on phylum Myxococcota, Proteobacteria, Firmicutes, and Bacteroidota, whereas soil AN and AP had negative impacts on phyla Bacteroidota, Actinobacteriota, Acidobacteriota, and Chloroflexi, with similar results at the genus level.

The main finding of the present study was that cattle manure deposition treatment increased relative abundance of bacteria known to be beneficial. Rhizosphere beneficial bacteria taxa are important links between plants and soil, not only reflecting soil nutrient status but also affecting plant growth (De Araujo et al., 2019). Proteobacteri, Bacteroidetes, and Firmicutes were present in maize, barley, cotton, and wheat rhizosphere (Peiffer et al., 2013; Guo Z. et al., 2019). Similarly, they were also the dominant bacterial phyla in oat rhizosphere, indicating they might be the core microbiome in most plant rhizosphere. Furthermore, cattle manure increased the relative abundance of Proteobacteria, Firmicutes, and Bacteroidota in oat rhizosphere when compared to control treatment at 15 and 66 days after sowing. Proteobacteria and Firmicutes are common in fecal matter and promoted the mineralization of complex organic compounds, reducing the C skeleton required for amino acid formation (Dai et al., 2018). Bacteroidota are important contributors to N and P turnover in the soil (Yousuf et al., 2012). At the genus level, *Pseudoxanthomonas* and *Pseudomonas* (bacterial Pseudomonadales order) were significantly enriched in oat rhizosphere in the present study following the application of manure. Furthermore, *Pseudoxanthomonas* and *Pseudomonas* genera directly solubilized phosphate and had a synergistic effect on promoting plant growth in soil (Bautista-Cruz et al., 2019; Li et al., 2021), protecting against pathogens (De Corato et al., 2020).

Here, we extended previous findings on fertilization-induced bacterial taxonomic changes to the functional level. It was reported that autotrophic nitrification increased after manure or inorganic N application in loamy (Tian et al., 2015) and neutral soils (Shen et al., 2012). However, nitrification was decreased by N inputs in black soil (Zhao et al., 2020). In that regard, ammonia-oxidizing bacteria (AOB) was suppressed as ionization of ammonia to ammonium was promoted by black soil acidification under fertilization (Zhang et al., 2012). A similar result was observed in the present study, indicating that functional bacteria related to nitrification were decreased by cattle manure, with its high concentrations of TN and AN. Cattle manure significantly increased the relative abundance of chemoheterotrophic and aerobic chemoheterotrophic communities, as they were positively correlated with N-acetyl-glucosaminidase activity, described as N acquisition enzyme (Wahdan et al., 2021).

#### Fungal Community Composition and Functional Groups

Cattle manure deposition treatment and plant growth stages altered the fungal community. Generally, Ascomycota is a dominant phylum in oat rhizosphere soil and decomposes organic matter in livestock manure (Yao et al., 2017). Similarly, in the present study, Ascomycota was dominant in the oat rhizosphere, but after the deposition of cattle manure, its relative abundance was much lower at 15 days and 66 days after sowing. Inexplicably, Ascomycota were considered key decomposers in agricultural soils and correlated with relatively high N content and availability (Paungfoo-Lonhienne et al., 2015). Perhaps the reduction of relative abundance of Ascomycota in the present study was due to available nutrients exceeding the tolerance limit of Ascomycota. Furthermore, Basidiomycota, another dominant decomposer in plant rhizosphere soil (Wang et al., 2018), decomposed organic matter and released N and P (Osono and Takeda, 2013). In the present study, the addition of manure increased Basidiomycota abundance at 15 days and 66 days after sowing.

More importantly, cattle manure deposition treatment reduced the community of fungi potentially pathogenic to oat. A previous study showed that cattle manure has a strong suppressive effect on soil pathogenic fungal growth and may regulate antagonistic microbial groups (Sun et al., 2016). In the present study, cattle manure deposition treatment decreased microbial groups related to pathotroph but increased beneficial groups of saprotroph and symbiotroph. It is well documented that high nutrient availability e.g., manure, inhibits the growth of pathogenic microbes (Mandavgane and Kulkarni, 2020) due to systemic acquired resistance (De Corato, 2020; Silva et al., 2022). In the present study, almost all fungal genera of the top eight dominant fungi were higher in the control treatment compared to cattle manure deposition treatment. Several of these fungi, e.g., genus *Alternaria* and *Fusarium* are soil-borne fungal pathogens with specific microbiomes in the rhizosphere (Sun et al., 2016). Plant diseases seriously hinder plant production and limit nutrients derived from plants (Raaijmakers et al., 2009).

## Conclusion

Deposition of cattle manure strongly promoted soil nutrient content and oat biomass. Ultimately 63.05% of fecal N and 51.41% of fecal P of cattle manure returned to the soil, whereas 11.79% of fecal N and 7.89% of fecal P were absorbed by oats throughout the entire growth period (80 days after sowing). The higher oat biomass following the application of manure was closely related to changes in root morphology and microbial community composition in oat rhizosphere soil. Cattle manure increased root length, surface, and volume of oats at all diameter classes, especially root length <0.2 mm diameter. In addition, cattle manure increased the beneficial microbiome, e.g., Proteobacteria, Bacteroidota, and Firmicutes bacteria, and suppressed soil fungal pathogens.

## Data Availability Statement

The datasets presented in this study can be found in online repositories. The names of the repository/repositories and accession number(s) can be found below: NCBI BioProject – PRJNA831303 and PRJNA830643.

## Author Contributions

RZ designed the experiments, provided the financial support, and helped perform the analysis with constructive discussions. CZ performed the experiments, analyzed the data, and wrote the manuscript. JH, QL, and YF helped revise the language of the manuscript. DL and ZL provided laboratory apparatus. All authors contributed to the article and approved the submitted version.

## Conflict of Interest

The authors declare that the research was conducted in the absence of any commercial or financial relationships that could be construed as a potential conflict of interest.

## Publisher’s Note

All claims expressed in this article are solely those of the authors and do not necessarily represent those of their affiliated organizations, or those of the publisher, the editors and the reviewers. Any product that may be evaluated in this article, or claim that may be made by its manufacturer, is not guaranteed or endorsed by the publisher.
