# Supplementary material for: Transfer of Nitrogen and Phosphorus From Cattle Manure to Soil and Oats Under Simulative Cattle Manure Deposition
Source: Front Microbiol. 2022 Jun 14;13:916610. doi: 10.3389/fmicb.2022.916610 (PMC9238326; doi:10.3389/fmicb.2022.916610)
Supplement: Supplementary file 1 [file Table_1.DOCX]

**Supplementary TABLE 1 |** Nutrient content of basic soil and cattle manure.

| Items | Nutrient content | |  |  |  |  |  |
| --- | --- | --- | --- | --- | --- | --- | --- |
|  | W | WC | SOC | TN | AN | TP | AP |
| Soil | 6.0 kg/pot | / | 13.69 g/kg | 0.922 g/kg | 148.2 mg/kg | 0.358 g/kg | 13.21 mg/kg |
| Cattle manure | 0.5 kg/pot | 780g/kg | 490.16 g/kg | 20.34 g/kg | 13.82 g/kg | 9.00 g/kg | 1.94 g/kg |

*Note: W, weight; WC, water content; SOC, soil organic carbon; TN, total nitrogen; AN, available nitrogen; TP, total phosphorus; AP, available phosphorus*.

**Supplementary TABLE 2 | The data evaluation of sample sequencing by 16S rRNA**

| Sample | Raw Tags | Clean Tags | Effective Tags | AvgLen (bp) | GC (%) | Effective (%) |
| --- | --- | --- | --- | --- | --- | --- |
| CON/TS | 79,697 | 79,691 | 77,996 | 417 | 56.01 | 97.87 |
| CON/TS | 79,648 | 79,639 | 78,322 | 420 | 57.03 | 98.34 |
| CON/TS | 79,598 | 79,590 | 78,096 | 416 | 56.09 | 98.11 |
| CON/TS | 79,818 | 79,811 | 79,349 | 419 | 57.66 | 99.41 |
| CON/TS | 80,093 | 80,082 | 79,796 | 417 | 57.16 | 99.63 |
| CON/TS | 80,319 | 80,288 | 79,414 | 419 | 57.04 | 98.87 |
| CMD/TS | 79,834 | 79,822 | 78,759 | 420 | 57.66 | 98.65 |
| CMD/TS | 80,395 | 80,378 | 79,239 | 419 | 57.67 | 98.56 |
| CMD/TS | 80,070 | 80,059 | 79,280 | 420 | 57.07 | 99.01 |
| CMD/TS | 79,950 | 79,932 | 79,126 | 420 | 58.22 | 98.97 |
| CMD/TS | 80,240 | 80,201 | 79,727 | 418 | 57.96 | 99.36 |
| CMD/TS | 79,640 | 79,624 | 78,809 | 419 | 57.91 | 98.96 |
| CON/TS | 80,281 | 80,278 | 79,415 | 416 | 56.21 | 98.92 |
| CON/TS | 79,976 | 79,963 | 78,455 | 418 | 56.74 | 98.10 |
| CON/TS | 79,943 | 79,928 | 79,165 | 419 | 56.99 | 99.03 |
| CON/TS | 79,837 | 79,833 | 78,287 | 415 | 55.81 | 98.06 |
| CON/TS | 79,510 | 79,506 | 78,611 | 418 | 57.53 | 98.87 |
| CON/TS | 80,035 | 80,025 | 79,430 | 418 | 57.42 | 99.24 |
| CMD/TS | 79,793 | 79,774 | 78,584 | 421 | 56.99 | 98.48 |
| CMD/TS | 80,188 | 80,168 | 79,114 | 420 | 57.67 | 98.66 |
| CMD/TS | 80,155 | 80,135 | 78,612 | 420 | 57.46 | 98.07 |
| CMD/TS | 80,131 | 80,126 | 79,528 | 420 | 57.47 | 99.25 |
| CMD/TS | 80,299 | 80,282 | 79,554 | 420 | 57.53 | 99.07 |
| CMD/TS | 79,799 | 79,782 | 78,851 | 420 | 57.64 | 98.81 |

*Note: TS, plant trefoil stage (15 days after sowing); PS, plant pustulation stage (66 days after sowing). CON, control; CMD, cattle manure deposition.*

**Supplementary TABLE 3 |** The data evaluation of sample sequencing by ITS rRNA

| Sample | Raw Tags | Clean Tags | Effective Tags | AvgLen (bp) | GC (%) | Effective (%) |
| --- | --- | --- | --- | --- | --- | --- |
| CON/TS | 79,265 | 66,902 | 66,867 | 313 | 52.75 | 84.36 |
| CON/TS | 79,750 | 62,337 | 62,221 | 349 | 48.4 | 78.02 |
| CON/TS | 80,014 | 63,080 | 63,025 | 371 | 52.0 | 78.77 |
| CON/TS | 79,921 | 66,521 | 66,515 | 294 | 48.37 | 83.23 |
| CON/TS | 80,272 | 62,031 | 62,012 | 334 | 49.07 | 77.25 |
| CON/TS | 80,191 | 70,730 | 70,376 | 274 | 49.88 | 87.76 |
| CMD/TS | 80,484 | 62,027 | 61,063 | 292 | 46.98 | 75.87 |
| CMD/TS | 79,715 | 52,466 | 52,380 | 299 | 50.16 | 65.71 |
| CMD/TS | 80,519 | 61,007 | 60,888 | 305 | 47.53 | 75.62 |
| CMD/TS | 80,154 | 67,654 | 67,649 | 277 | 51.27 | 84.4 |
| CMD/TS | 79,921 | 62,264 | 61,997 | 291 | 49.75 | 77.57 |
| CMD/TS | 79,863 | 61,075 | 61,016 | 294 | 48.25 | 76.4 |
| CON/TS | 79,830 | 63,516 | 63,418 | 330 | 48.07 | 79.44 |
| CON/TS | 80,267 | 63,266 | 63,232 | 261 | 46.0 | 78.78 |
| CON/TS | 79,782 | 61,864 | 61,590 | 298 | 49.1 | 77.2 |
| CON/TS | 79,895 | 66,395 | 66,262 | 291 | 47.89 | 82.94 |
| CON/TS | 80,112 | 62,613 | 62,483 | 293 | 47.84 | 77.99 |
| CON/TS | 80,090 | 66,958 | 66,811 | 299 | 46.97 | 83.42 |
| CMD/TS | 79,753 | 65,092 | 65,075 | 294 | 49.09 | 81.6 |
| CMD/TS | 79,904 | 61,471 | 61,393 | 310 | 49.29 | 76.83 |
| CMD/TS | 79,644 | 51,246 | 51,148 | 329 | 49.44 | 64.22 |
| CMD/TS | 80,055 | 62,758 | 62,726 | 309 | 49.06 | 78.35 |
| CMD/TS | 79,761 | 63,448 | 63,256 | 305 | 49.37 | 79.31 |
| CMD/TS | 79,861 | 62,391 | 62,389 | 312 | 46.99 | 78.12 |

*Note: TS, plant trefoil stage (15 days after sowing); PS, plant pustulation stage (66 days after sowing).* *CON, control; CMD, cattle manure deposition.*

**
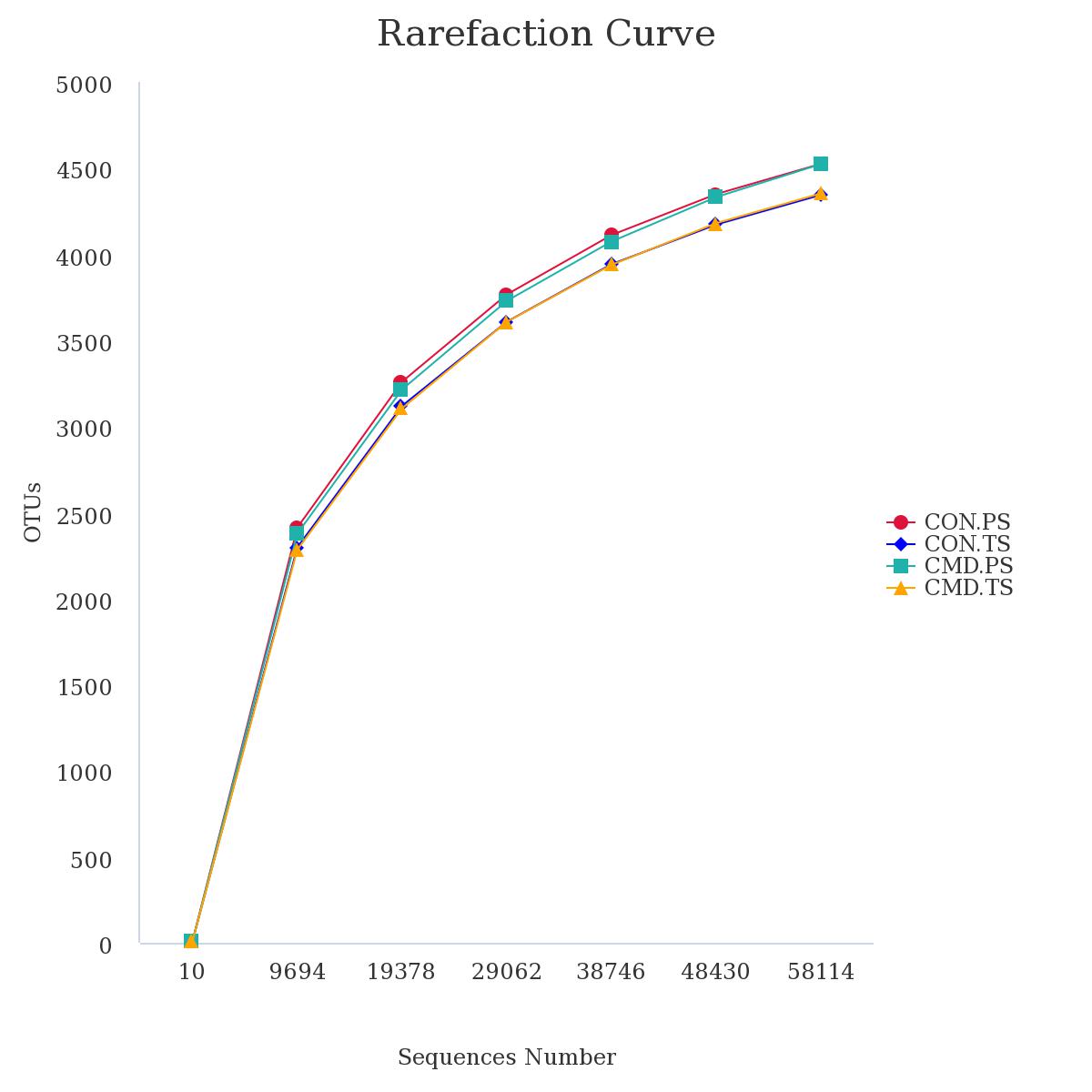
**

**Supplementary FIGURE 1 |** The rarefaction curves showing the relationship between the sequencing depth and the number of OTUs in each soil sample for bacteria

*Note: TS, plant trefoil stage (15 days after sowing); PS, plant pustulation stage (66 days after sowing).* *CON, control; CMD, cattle manure deposition.*


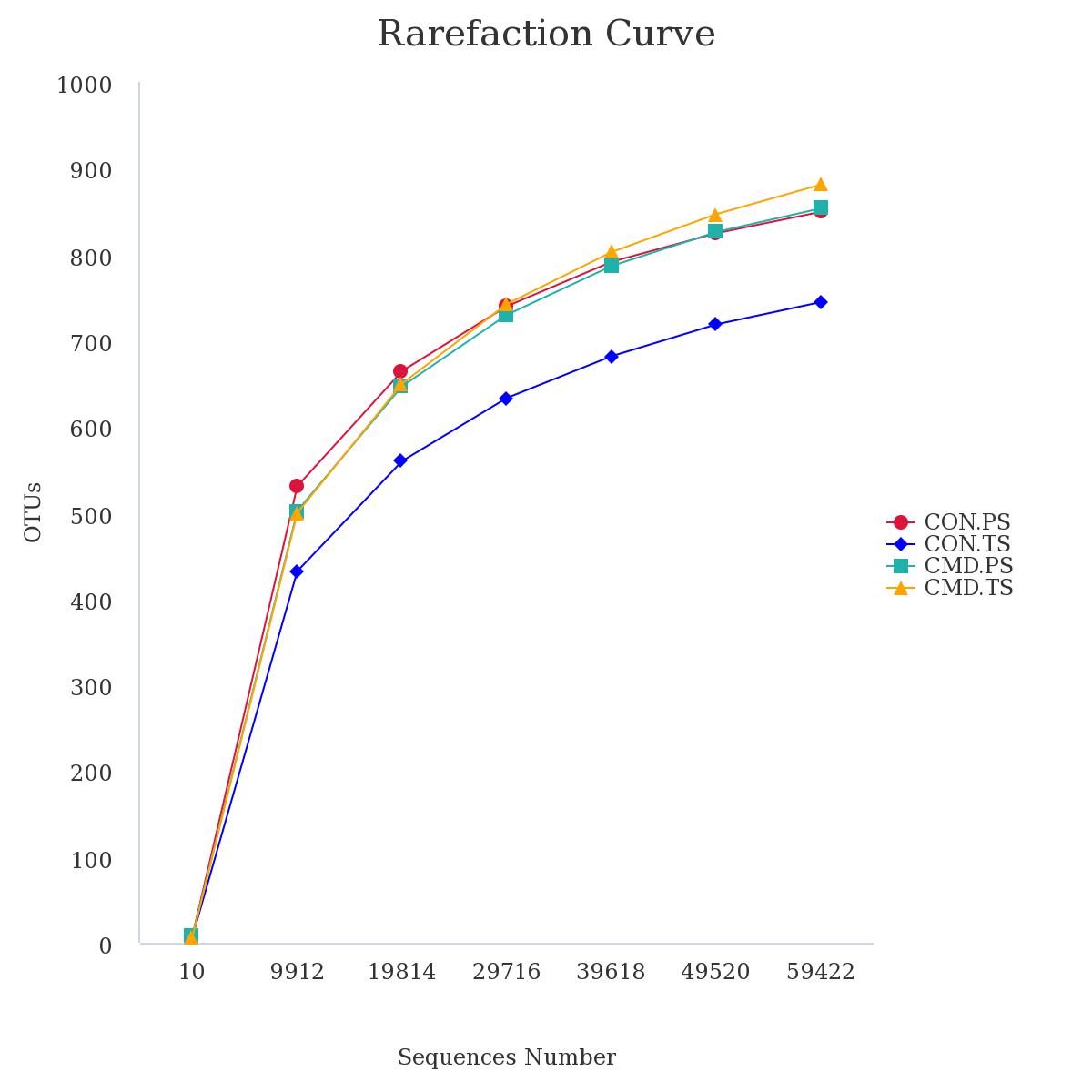
. **Supplementary FIGURE 2 |** The rarefaction curves showing the relationship between the sequencing depth and the number of OTUs in each soil sample for fungi

*Note: TS, plant trefoil stage (15 days after sowing); PS, plant pustulation stage (66 days after sowing).* *CON, control; CMD, cattle manure deposition.*

**
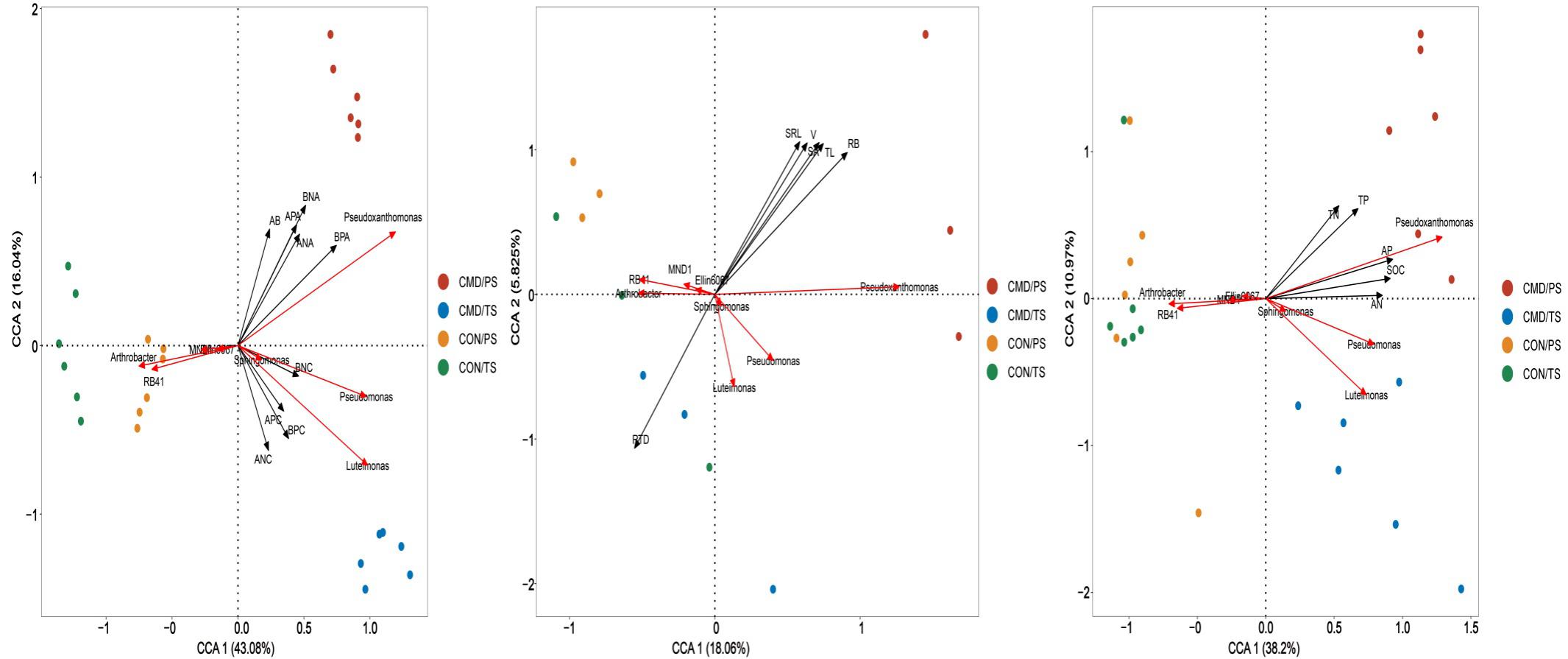
**

c

b

a

**Supplementary FIGURE 3 |** Canonical correspondence analysis (CCA) showing bacterial community composition at genus level as affected by biomass and nutrient absorption (a), and root traits (b) soil chemical property (c). Microbial community composition located in the forward direction of the arrow of the environmental factor implies positive relationship between the microbial community composition and the environmental factor, and vice versa. *Note: TS, plant trefoil stage (15 days after sowing); PS, plant pustulation stage (66 days after sowing); AB, aboveground biomass; RB, below-ground biomass; ANC, above-ground N content; BNC, below-ground N content; APC, above-ground P content; BPC, below-ground P content; ANA, above-ground N accumulation; BNA, below-ground N accumulation; APA, above-ground P accumulation; BPA, below-ground P accumulation. CON, control; CMD, cattle manure deposition. RL, Root length; SA, root surface area; V, root volume, SRL, specific length; RTD, root tissue density; SOC, soil organic carbon; TN, total nitrogen; AN, available nitrogen; TP, total phosphorus; AP, available phosphorus. Data represents mean ± SE (n = 3). The data was based on a pot experiment in one year. All parameters were analyzed with SPSS version 16.0 (SPSS Inc. Chicago, IL, USA)*

**
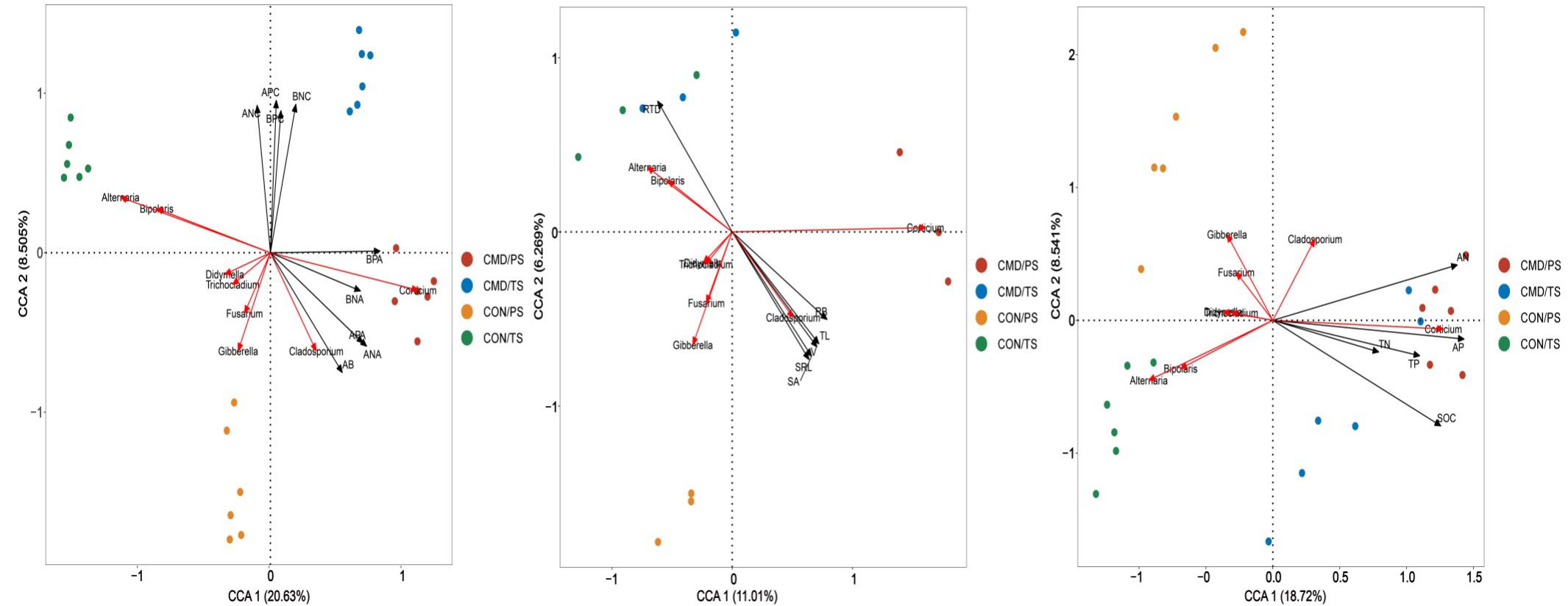
**

c

b

a

**Supplementary FIGURE 4 |** Canonical correspondence analysis (CCA) showing fungal community composition at genus level as affected by biomass and nutrient absorption (a), and root traits (b) soil chemical property (c). Microbial community composition located in the forward direction of the arrow of the environmental factor implies positive relationship between the microbial community composition and the environmental factor, and vice versa. CON, control; CMD, cattle manure deposition.
